# The Influence of Classroom Size and Window View on Young Children’s Executive Functions and Physiological Responses, Based on VR Technology

**DOI:** 10.3390/bs13110936

**Published:** 2023-11-16

**Authors:** Kijoo Cha

**Affiliations:** Department of Early Childhood Education, Gachon University, Seongnam-si 13120, Republic of Korea; kijoocha@gachon.ac.kr

**Keywords:** virtual reality, kindergarten, executive functions, classroom size, window view

## Abstract

Despite the increasing enrollment of young children in childcare institutes, there have been few empirical studies on the effects of spatial elements on their development. This study explored the impact of preschool classroom size (large vs. small) and window view (natural vs. built environment) on young children’s executive functions and physiological stress responses, using cortisol and heart rate variability (HRV) as indicators and employing virtual reality (VR) technology. Out of 144 participants aged 61–85 months, three were excluded due to missing values and outliers. Executive function tests were administered, and saliva samples were collected before and after VR exposure; HRV data were gathered during the experience. ANCOVA results indicated significant improvements in cognitive flexibility, as measured based on the Dimensional Change Card Sorting task, in the large classroom condition, and a marginally significant decrease in visuo-spatial working memory, as measured with the Corsi block task, in the small classroom condition. The classroom size conditions did not significantly differ in cortisol response, but the large classroom condition showed marginally significant HRV indices, suggesting increased relaxation. No significant effects on executive functions or physiological responses were found in either window view condition. Overall, the findings suggest that classroom size may influence young children’s cognitive flexibility.

## 1. Introduction

Early childhood is characterized by the highest levels of brain plasticity in response to external stimuli [1]. In more than two-thirds of OECD countries, 80% or more of three-to-five-year-old children participate in some sort of early-childhood education and care (ECEC) programs [2]. It is therefore essential to provide high-quality ECEC environments. Indeed, many studies have revealed links between the quality of ECEC environments and young children’s health and cognitive and socio-emotional development [3,4,5]. However, most of these prior studies have relied on comprehensive quality indices, which provide overall scores for physical, social, and educational dimensions, without determining the impact of specific individual components of the various sub-dimensions.

In addition, despite accumulating evidence that spatial elements in a building’s interior are significantly associated with individuals’ cognitive, emotional, and physiological functioning, including educational settings [6,7,8,9,10,11], few studies have examined how individual spatial characteristics of classrooms influence the behavior and development of young children [12,13,14]. Examples include Read et al. (1999) [13], who found an association between the ceiling height and wall color of an ECEC classroom and children’s cooperative behaviors, and Abbas et al. (2016) [12], who linked the spatial arrangement of areas in a preschool classroom with prosocial behaviors.

Thus, given the dearth of studies concerning the effects of specific spatial elements in educational settings on young children’s development, the current study focuses specifically on the extent to which natural views and a larger classroom size, both of which are well known to provide cognitive and emotional benefits to older students and adults, influence the cognitive functioning and stress responses of preschool-aged children.

### 1.1. The Impact of Visual Exposure to Nature on Humans

It is well known that the greenness of interior spaces, including access to a natural view, indoor plants, and images of nature (e.g., photos, posters), can positively influence human cognition (e.g., attention, memory, creativity) and emotions (e.g., stress reduction or recovery, feelings of comfort) [9,10,15,16,17,18]. These findings have been theoretically grounded in the stress reduction theory (SRT) [19] and the attention restoration theory (ART) [17,20], both of which concern the positive effects of interacting with nature. The SRT primarily centers on the idea that exposure to natural environments can help to reduce stress and promote relaxation. It proposes that natural environments provide a sense of tranquility and emotional restoration, which leads to a decline in stress-related physiological responses, such as cortisol levels, heart rate, and blood pressure. In line with the SRT, the ART focuses particularly on the cognitive benefits of nature, especially its role in restoring and replenishing attentional capacities and thereby enhancing cognitive performance and mental clarity [17,20]. The theory suggests that exposure to nature can improve attention and cognitive functioning after tasks requiring directed focus, since nature, as an inherently unique form of “soft fascination”, captures human attention effortlessly and allows the mind to rest and recover from attention depletion and mental fatigue [17,20]. Despite of a great deal of prior studies evidencing both theories, most of these studies have focused on adult participants in the workplace or in hospitals, neglecting the impact of school buildings on children, and on young children in particular.

In recent decades, more studies have recognized these gaps in the literature and attempted to replicate known results in educational settings by investigating the association between visual access (via windows) to green landscapes and students’ cognitive and emotional wellbeing [8,9,21,22]. For instance, Li and Sullivan (2016) [9] investigated the effects of classroom window views on high-school students’ attention restoration and stress recovery. Based on a randomized controlled experiment, they allowed students to engage in class activities and take breaks in three different conditions: with no window, with a natural view (characterized by trees, grass, bushes, or lakes), and with a built-environment view (characterized by buildings, streets, or parking lots). Students exposed to the nature view condition performed significantly better on break-time attention tests (Digit Span Forward and Backward) than those in the built view condition. Another study involving high-school students [21] showed similar results: students who had more visual access to trees and shrubs through classroom and cafeteria windows tended to have higher standardized test scores, graduation rates, and four-year college plans; fewer engaged in criminal activities. Another study of college classrooms showed that students with a natural view during a one-semester course recorded higher course satisfaction ratings and attained better grades at the end of the semester than those whose view was a concrete wall [8]. Natural views from university dormitory windows have also been shown to have a positive effect on residents’ directed attention scores [23]. In addition, exposure to natural views, indoor plants, and the color green have been found to boost creativity among business students [22].

However, few studies have examined the beneficial effects of natural elements on younger students; only a couple of studies have considered elementary school students (e.g., [24,25,26]. For instance, in Lindemann-Matthies et al. (2021) [25], associations between natural/built window views and fourth graders’ self-reported wellbeing and standardized attention and concentration test scores have been investigated. Students with greener classroom window views have reported to be more focused and less stressed, but their performance on an attention and concentration test was not influenced by natural classroom window views, a finding that differs from those of previous studies concerning older age groups. In contrast, some studies have suggested similar effects found in older student populations, albeit not specifically related to window views: their findings concern natural objects, including the impact of indoor plants on fifth, sixth, and seventh graders [26] and sixth and seventh graders [24]. Children in an indoor plant condition (a green wall) performed better on a selective attention test, although their subjective levels of emotional and social wellbeing did not differ significantly from those of control group participants [26]. In another study, sixth and seventh graders in a classroom in which three indoor plants had been present for six weeks scored higher in reading, writing, and mathematics [24].

Despite a wealth of evidence to support the argument that visual exposure to nature can enhance students’ cognitive performance and reduce stress, gaps in the literature remain. First, there has been a definite lack of research on preschool-aged children in ECEC settings. Next, very few studies have used objective physiological measures in place of subjective feelings (e.g., reported stress levels) or randomized controlled designs to infer causality among variables of interest [9]. This lack of randomized experimental studies likely reflects the practical difficulties involved in finding or building two or more classrooms that differ only in their visual exposure to greenness.

To address such difficulties, some studies have used VR technology. Palanica et al. (2019) [10] compared the effects of natural versus urban views on human creativity using two-dimensional (2D) videos, three-dimensional (3D) VR, and real-life outdoor settings. The participants performed better on creativity tests in natural environments than in urban ones, when exposed to natural and urban conditions for four minutes via a 2D mobile tablet and a 3D VR headset, respectively. Zhou and Ergan (2019) [27] investigated the impact of interior features in a corridor on cognitive tasks (e.g., word memory and object search) using VR technology. They created two versions of a virtual space (positive vs. negative), which differed only in terms of the presence and size of windows and the presence of natural light and natural views. The positive environment had all of these features, while the negative environment had none. The participants stayed in each space for ten minutes and were then tested immediately on a recall task for five minutes. In the positive environment, participants exhibited more focused attention, finished the tasks within a shorter period of time, and recalled more words than they did in the negative environment. Although the study collected data based on objective measures, the effects of individual elements of the positive environment (i.e., window, lighting, and outside views) were measured together as a whole, making it impossible to determine how much of the observed effects could be attributed to each individual factor.

### 1.2. Impact of Room Size on Humans

Compared with research on the impact of natural elements, only a few studies have measured the effects of room size on individual performance or wellbeing. Recently, Cruz-Garza et al. (2022) [28] investigated the impact of room size and window placement on the cognitive performance of college students (i.e., Stroop, digit span, Benton, visual memory, and arithmetic tests) using VR technology and electroencephalography (EEG). EEG data were collected from participants who wore VR headsets and took five cognitive tests in four different versions of a virtual classroom: (1) with no window, (2) with a wider space and no window, (3) with one window on the left-hand wall, and (4) with two windows at the front. Contrary to the authors’ expectations, no significant test score differences were found across these VR classrooms; however, consistent patterns were detected in the EEG features. These results imply that classroom size is likely to cause changes in the transfer of information across brain regions, suggesting that classroom design can affect students’ cognitive functioning.

In contrast to the dearth of literature on the impact of classroom size, a larger group of studies have investigated class size (the number of students), overall finding a positive association between large class sizes and low academic achievement, and vice versa [29,30]. Another line of relevant research concerns the space allocated to each student, with the appropriate classroom size being clearly related to the number of children in one class. Maxwell (2003) [31] has distinguished density by class size (students per class) from density by room size (space per person), labelling the former social density and the latter spatial density. As discussed above, far fewer studies have considered spatial density than social density in educational settings. Of these, Maxwell (2003) [31] has examined the effects of both types of density among elementary school children (second and fourth graders). This study found that, while spatial density mattered more for girls, social density mattered more for boys. For girls, the smaller the space per student, the lower the academic achievement; for boys, the more children per class, the more disturbing behaviors emerged in class (e.g., hyperactivity, hostility, and anxiety). These results suggest that classroom size matters as much as class size [31]. Regarding ECEC settings, earlier studies have found that higher levels of spatial density (more children in a given physical space) predict fewer social and cooperative interactions between peers (more solitary and parallel play than group play), more aggressive behaviors, and less gross motor play [32,33,34]. An increase in off-task time is also associated with a higher ratio of children to activity areas [35]. In sum, classroom size is likely to affect children’s stress levels and cognitive and behavioral regulation [36]. However, no single study has investigated the impact of classroom size itself, independent of social crowding effects, on young children’s cognitive functioning and stress responses. Furthermore, as in the case of the window view literature, no randomized experimental studies have been carried out to determine the effects of room size.

In sum, this review of the previous literature suggests that a view of green nature and a spacious classroom can reduce stress and enhance cognition. As discussed above, however, there are gaps in the literature concerning both window views and room sizes. In this context, the current study uses a randomized experimental design (with VR technology) to determine whether the view and size of an ECEC classroom have a significant impact on preschool children’s executive functions (EFs). This study also investigates whether different VR conditions can generate discrepancies in children’s physiological indices of stress and resilience, such as cortisol levels and heart rate variability (HRV). This inquiry was grounded in existing research that demonstrates the significant effects of VR environments on young children’s cognitive and behavioral responses [37,38]. Executive functions, which comprise working memory, inhibitory control, and cognitive flexibility, are known to administer top-down mental processes that are crucial for high-order cognition and are negatively affected by stress [39,40]. Given that visual exposure to nature is associated with positive cognitive functioning (e.g., memory, attention) and spatial density is related to mild stress [41] and attentional overload in adults [42], this study argues that a window view (of a natural vs. a built environment) and classroom size (large vs. small) may also influence children’s executive functions (EFs), and indicators of stress levels and stress resilience (cortisol and HRV). Heart rate variability, as part of the interdependent regulatory system (autonomic nervous system, ANS) can help individuals adapt to environments [43]; it also reflects physiological stress levels [44] and is positively associated with EF [45,46]. Thus, children may display distinctive HRV responses under different VR conditions.

### 1.3. Research Questions and Hypotheses

To examine whether the size and window views of VR classrooms influence young children’s performance on EF tasks and physiological responses (i.e., cortisol level and HRV), this study proposes the following research questions (RQs):

RQ1. Does classroom size (large vs. small) influence young children’s performance in EF tasks or their physiological responses (i.e., cortisol and HRV)?
  RQ1.1. Does classroom size (large vs. small) influence young children’s performance in EF tasks?
  RQ1.2. Does classroom size (large vs. small) influence young children’s physiological responses (i.e., cortisol and HRV)?
RQ2. Does window view (nature vs. built) influence young children’s performance in EF tasks or their physiological responses (i.e., cortisol and HRV)?
  RQ2.1. Does window view (nature vs. built) influence young children’s performance in EF tasks?
  RQ2.2. Does window view (nature vs. built) influence young children’s physiological responses (i.e., cortisol and HRV)?

Based on the previous literature, it was hypothesized that children in the larger classroom and nature view conditions (experimental groups) would exhibit higher performance in EF tasks, lower cortisol levels, and higher-level results in HRV indices (better stress resilience) than those in the smaller classroom and built view conditions (comparison groups).

## 2. Materials and Methods

### 2.1. Participants

One hundred forty-four children and their mothers, living in the capital area of South Korea, were recruited for this study. Children were eligible to participate if they (1) had never had a neurological disorder or mental health condition (e.g., schizophrenia, ADHD, epilepsy) or family members with such a disorder or condition; (2) had never been medicated due to any other disease; (3) were not color blind and had corrected vision above 0.8; (4) were five or six years old at the point of participation (i.e., born in 2015 or 2016); and (5) had previously had a VR experience without encountering any abnormal symptoms. Although a short exposure to VR has been found to be harmless to young children [37,38], only five- or six-year-old children with pre-virtual experiences, in the final grade of pre-primary education, were recruited for safety reasons. To recruit participants, flyers were posted on websites known to attract frequent visits and participation from the mothers of preschool-aged children. In age, the participants ranged from 61 to 85 months (mean = 74.62, SD = 4.87); the group consisted of approximately 48.6% boys (70 boys and 74 girls). Each parent–child pair was offered approximately USD $50 for their participation, during which the child engaged in saliva collection, executive function (EF) tests, and virtual experience, while the parent completed a questionnaire regarding family socio-demographics and the child’s temperament. The experimental procedures, including the procurement of participant information sheets and consent forms, were approved by the University Bioethics Review Committee (Approval No. 1044396-202007-HR-138-04).

### 2.2. Procedures

#### 2.2.1. VR Development and Equipment

The Worldviz Vizard VR toolkit was used to create virtual preschool classrooms. A desktop computer rendered the scenes, while the immersive 3D preschool classroom was displayed on an HMD, the HTC Vive Pro Eye. The HTC Vive Pro Eye offers stereoscopic views and a 110° horizontal field of view, with a resolution of 2880 × 1600 per eye and a refresh rate of 90 frames per second (90 Hz). “Lighthouse sensors” built into the headset and the strap around each user’s head picked up infrared signals and tracked the user’s physical head movements and orientation. In conjunction with a gyroscope, accelerometer, and magnetometer, these enabled the headset to adjust to the virtual environment.

#### 2.2.2. VR Classroom Design Conditions

**Figure 1:**
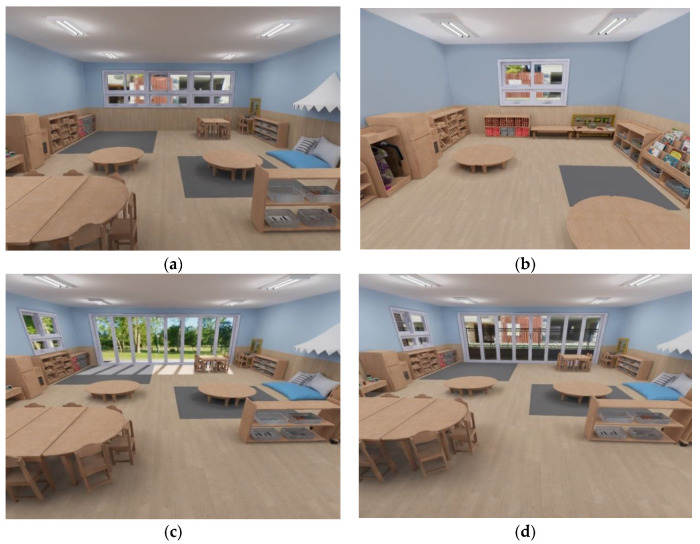
Two-dimensional captures of the four immersive 3D preschool classrooms: (**a**): large classroom; (**b**): small classroom; (**c**): nature view; (**d**): built view.

Two contrasting versions of the classroom were created for each of the two treatment variables (room size and window view) for a total of four virtual classroom conditions. To test the effect of the room size, large and small classrooms were created (Figure 1a,b). To test the effect of the window view conditions, classrooms with a built view (a concrete building and street) and a natural view (a field of grass and trees) were created (Figure 1c,d). The room sizes were all identical (except in the small-room condition) at 2.6 m (H) × 8.9 m (W) × 7 m (D). The program thus allocated 3.1 m^2^ per child (assuming a class size of 20), which is slightly over the average minimum standard for space per child in ECEC settings among OECD countries (2.9 m^2^ for kindergarten/preschool) [2]. The small classroom was 2.6 m (H) × 5.0 m (W) × 4.0 m (D), allocating 1 m^2^ per child (assuming a class size of 20); it was therefore about one third the size of the large classroom. For the window view condition, the classrooms were designed to be as spacious as the large classroom condition to avoid any confounding stress-related effects on the children’s cognitive and emotional functioning.

Across these virtual classrooms, all conditions, apart from the treatment variables (room size and window view) remained constant. In the classroom size conditions, however, the small room could not hold as many pieces of furniture or play materials as the large room. To minimize any interference with the children’s responses, the types and number of activity areas and essential materials were identical, with only peripheral furniture (mostly chairs) excluded from the small room version (Please see the Supplementary Material for the floor plans for each VR condition).

#### 2.2.3. Experimental Procedures

Parents, primarily mothers, registered to participate using Google Forms online. The applicants were grouped first by their children’s gender (male and female) and then randomly assigned to one of four virtual spaces. General information about the study was explained to parents over the phone (i.e., purpose, procedures, compensation), after which visits to the university laboratory were scheduled. On the day before the visit, the research team sent a text message to the parents, reminding them of the time and place and explaining what the child should avoid doing within an hour of his/her visit (e.g., chew gum, eat food, or wear lip balm, as these could affect the saliva and cortisol analysis). Cortisol levels change depending on diurnal rhythms, typically peaking at 8 AM and gradually declining by nighttime, with a couple of low surges midway through. To control for the effects of cortisol time dependency, the experiment was conducted at regular times for each condition throughout the data-collection period: at ten AM for the room size condition and at 2 PM for the window view condition. As data were collected during the COVID-19 pandemic, all participants wore masks for the experimental procedures. The temperature and humidity of the laboratory where the experiment was conducted were maintained throughout the data collection period.

On the day of the visit, each parent–child pair arrived at the university laboratory waiting room and received a second briefing on the experimental procedures. After re-confirming their consent to participate, the researcher led each pair to the next room for the experiment, giving them five minutes to familiarize themselves with and navigate the room freely. A research assistant then explained to the child that his/her mother would return to the waiting room, with the door left half open if s/he preferred it that way. While in the waiting room, the mother signed the consent form and completed a questionnaire about family socio-demographics and the child’s temperamental characteristics. The mother and child were not informed of their assigned condition until the end of the experiment or given any direction or information that could influence the study results, e.g., the reasons for designing four different versions of the virtual classroom.

When the mother left the room, a research assistant collected the child’s saliva and helped him/her strap a heart rate monitor around his/her chest. The child was then led to a computer in a corner of the room and asked to take computer-based EF tests, which took approximately 10–15 min to complete. After the pre-test, the child was guided to the center of the room, where a large square was drawn on the floor. Within this square, the child was free to move in immersive virtual reality, wearing a VR headset. At the start of the VR experience, s/he was guided to sit on a chair placed in the center of the square and fitted with the VR headset. Then, s/he was given one minute to look around a non-test VR environment (the hallway) to become accustomed to the VR equipment and to see whether the exposure caused any problems or uncomfortable feelings.

**Figure 2:**
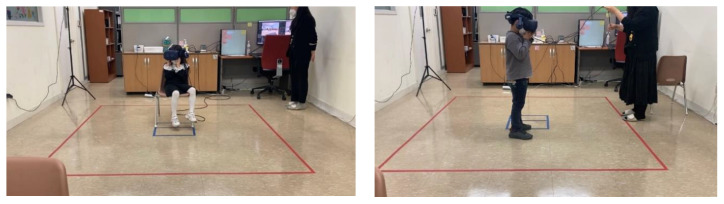
Participating children wearing the VR headset and exploring the VR classroom for ten minutes (five spent sitting and five spent walking around).

Next, the child experienced the VR classroom for ten minutes. As it was difficult for the children to sit still for ten minutes, they were allowed to move around for the last five, after spending the first five sitting and looking around the room (Figure 2). Adult participants in prior VR studies have generally been given four or five minutes and up to ten [10,27,28]; the current study set an exposure time of ten minutes, on the grounds that children might need longer to navigate and adjust to a new virtual setting and might also want to stay longer, given the novelty of the VR environment.

Throughout the procedures, a researcher checked each child’s physical reactions and asked him/her to mention any strange feelings or symptoms; the researcher also monitored what the child was seeing through the computer screen. As soon as the VR experience was over, computer-based EF tests were conducted (post test), and the child’s saliva was collected again. To prevent any practice effects, slight alterations were made to the contents of the post tests, while keeping the test levels the same (e.g., for digit span, alterations in digits (e.g., 5 → 3); for DCCS, substitutions in target objects (i.e., rabbit → flower); for Corsi block, adjustments in the block arrangement). Further information about these tasks can be found in the Measures section, which provides detailed descriptions about them. It took less than 60 min (one hour) to complete the entire procedure.

### 2.3. Measures

Socio-demographic information: Information was collected on the parents’ educational attainment, based on the following response options: (1) middle-school graduate, (2) high-school graduate, (3) 2- or 3-year college graduate, (4) 4-year college graduate, and (5) graduate-level degree(s). Monthly family income was measured using the following response options: (1) below $999; (2) $1000–$1999; (3) $2000–$2999); […], (9) $8000–$8999; (10) 9000-9999, and (11) more than $100,000.

Child Temperament: Parents completed the Children’s Behavior Questionnaire Short Form (CBQ), consisting of three subscales [47]: surgency (Cronbach’s alpha = 0.76), negative emotionality (Cronbach’s alpha = 0.66), and effortful control (Cronbach’s alpha = 0.75). The subscale items were measured using a seven-point Likert scale, with responses ranging from 1 (extremely untrue of my child) to 7 (extremely true of my child) with “not applicable” as an additional option.

Executive Function tasks: The following three widely adopted and developmentally sensitive EF tasks were administered on a computer to measure attentional control and different types of working memory (e.g., [48], which have been found to be affected by stress and concentration in the previous literature, as described earlier: (1) digit span forward (verbal working memory and attention), (2) Corsi block (visuo-spatial working memory and attention), and (3) Dimensional Change Card Sorting (DCCS) (attentional flexibility). In the digit span task, the children were asked to listen carefully to a random series of digits presented through the computer and then to repeat them orally in the order presented, which was entered into the computer by the researcher. The task began with two digits and gradually increased the number until the child failed to repeat one sequence in either trial. The longest sequences reproduced by the children were used as their digit span scores [49]. Next, the Corsi block-tapping task was administered and scored, in accordance with Kessels et al. (2000) [50]. Nine squares were positioned at random on the touch screen and the children were initially asked to watch a subset of the squares flashing in a predetermined order on the screen; they then tapped them in the same order. When the children answered twice correctly (two trials per level), the number of blocks they were asked to tap in sequence increased. By contrast, when they failed to reproduce the correct order in two out of three trials, the test ceased at that level. The children’s Corsi spans, or block spans (defined as the longest sequence a participant was able to reproduce via tapping, ranging from 0 to 9), were calculated and used in the analysis. The task protocol for the DCCS was based on Zelazo (2006) [51]. In this task, the children were asked to sort cards one at a time in one dimension (shape or color) by tapping the target cards in either category at the foot of the touch screen. Next, they were asked to switch and sort their cards in the other dimension. As the Korean children exhibited ceiling performance on the DCCS task [52] and the study participants were all five- to six-year-olds at the point of measurement, the border version was tested on every participating child. The “border version” introduces an adaptation to the sorting rules to increase task complexity, denoting a scenario where a child is directed to alternate between sorting rules for cards with and without borders. In this rendition, a child is presented with a collection of cards on the screen, comprising both bordered and non-bordered ones. They are then instructed to sort the non-bordered cards by color and the bordered cards by shape [51]. Correct scores on the DCCS (the number of correct answers in all trials) ranged from 0 to 36 (six trials (color), six trials (shape), and 12 trials (border)), which were used in the analysis.

Cortisol levels: As noted above, saliva samples were collected twice: before and after the EF tests and VR exposure. The collected samples were kept frozen until the analysis. The cortisol was analyzed using the Cortisol Saliva DSNOV20 kit (NovaTec, Stuttgart, Germany), which had a detection limit of 0.12 ng/ mL and inter-assay and intra-assay coefficients of variation of 8.3% and 10%, respectively.

Heart rate variability (HRV): HRV information was collected using a Polar H10 heart rate sensor (Polar, Finland) during a ten-minute VR exposure; only the first five minutes of information were analyzed in the current study because the HRV data had to be collected when the participants were breathing at a normal rate in a resting state and sitting upright without pacing [43]. Among the few indices of HRV that are widely used in the relevant literature, the current study reports SDNN, NN50, pNN50, and RMSSD [43,53]. First, SDNN stands for “Standard Deviation of Normal-to-Normal intervals”, where the normal-to-normal intervals refer to the time intervals between successive normal heartbeats on an electrocardiogram (ECG) signal [43]. In short, “SDNN” refers to the standard deviation of inter-beat intervals (IBIs), when abnormal beats have been removed. As the SDNN is thought to reflect the global activity of the ANS and relates to parasympathetic tone [43], higher values indicate a healthier body state, better resilience against stress, and better adaptability to the environment [54]. NN50 is the average number of successive normal sinus (NN) intervals that exceed 50 ms, and pNN50 is the proportion of NN50. RMSSD is defined as the root mean square of successive differences between normal heartbeats, indicating a beat-to-beat variance in heart rates. Since NN50, pNN50, and RMSSD are highly associated with each other (see Table 1) and also with parasympathetic tone [53], higher values indicate a more relaxed state and lower levels of stress in the body.

### 2.4. Analyses

Prior to addressing the research questions, descriptive statistics and correlations between the main variables were examined. During this process, two children were found to have unusually high cortisol levels (pre-level: M = 26.57 nmol/L, SD = 1.44; post-level: M = 33.37 nmol/L, SD = 14.08), compared to the rest of the sample (pre-level: M = 6.48 nmol/L, SD = 2.93; post-level: M = 5.82 nmol/L, SD = 2.47) and exhibit an abnormal pattern of change showing more elevated values at the post measurement. Although no standard range of diurnal cortisol specifically for non-clinical young children has been proposed, these two children’s cortisol levels were substantially higher even when compared to the findings from Davis et al. (2002) [55]. In that study, with 5- to 6-year-old children (n = 52), the levels were 0.71 µg/dL (equivalent to 19.59 nmol/L) around 8 am (the peak point) and 0.25 µg/dL (equivalent to 6.90 nmol/L) at approximately 4:30 pm. In addition, one child’s saliva sample was missing. Three children (one boy and two girls) were therefore excluded from the analyses. In total, 141 children were included in the analyses, with 69 children in the room size condition (large: n = 34 (17 boys and 17 girls), small: n = 35 (18 boys and 17 girls)), and 72 children in the window view condition ((nature: n = 38 (18 boys and 20 girls); built: n = 34 (16 boys and 18 girls)).

To check randomization, independent *t*-tests were conducted between the experimental and comparison groups (i.e., large vs. small and nature vs. built) across the children’s background and temperament variables and pre-test scores on the EF tests. Unequal variances between the two groups were adjusted in the statistical analyses. First, in relation to the room size condition, no significant differences were detected between the large and small classroom groups: child age (in months): *t* = −0.89, *p* = 0.18; years of childcare experience: *t* = −0.02, *p* = 0.49; father’s age: *t* = 1.54, *p* = 0.93; mother’s age: *t* = 1.24, *p* = 0.89; surgency: *t* = 0.70, *p* = 0.75; negative emotionality: *t* = 0.53, *p* = 0.70; effortful control: *t* = −0.79, *p* = 0.22; child birth order: χ^2^ = 1.71, *p* = 0.64; child sex: χ^2^ = 0.01, *p* = 0.91; father’s education: χ^2^ = 3.35, *p* = 0.50; mother’s education: χ^2^ = 6.72, *p* = 0.08; family income: χ^2^ = 12.65, *p* = 0.18. In relation to the pre-test EF scores, a significant difference was found only in the Corsi block task for the room size condition: digit span: *t* = −0.55, *p* = 0.29; Corsi block: *t* = 1.71, *p* = 0.95; DCCS: *t* = 0.77, *p* = 0.78.

Similarly, in relation to the window view condition, there were no significant differences between the built and nature view groups across the socio-demographic and temperamental variables, apart from a higher level of child surgency in the nature view group (*t* = −1.83, *p* = 0.04): child age (in months): *t* = −1.26, *p* = 0.11; years of childcare experiences: *t* = 0.05, *p* = 0.52; father’s age: *t* = 0.14, *p* = 0.56; mother’s age: *t* = −0.06, *p* = 0.47; negative emotionality: *t* = −1.23, *p* = 0.11; effortful control: *t* = 0.61, *p* = 0.73; child birth order: χ^2^ = 2.85, *p* = 0.42, child sex: χ^2^ = 0.00, *p* = 0.98; father’s education: χ^2^ = 1.46, *p* = 0.69; mother’s education: χ^2^ = 0.93, *p* = 0.82; family income: χ^2^ = 10.64, *p* = 0.30. Regarding the scores on the pre-EF tests, no significant difference was found: digit span: *t* = −0.94, *p* = 0.18; Corsi block: *t* = −1.27, *p* = 0.10; DCCS: *t* = −0.20, *p* = 0.42.

An ANCOVA was conducted to determine the effects of the VR conditions (namely classroom size and window view) on the post-EF scores and post-cortisol levels, taking into account the respective pre-measurement values as covariates. The choice of ANCOVA over MANCOVA was made due to the very weak or no correlations among different EF measures (Table 1), as MANCOVA is more suitable for cases with stronger multivariate relationships [56]. The children’s temperamental and sociodemographic characteristics, including child sex, were not considered in the main analyses as covariates because no significant associations were found in the correlational analysis (Table 1). As for the HRV, to assess potential differences between the experimental and comparison groups while avoiding multicollinearity, independent *t*-tests were employed while accounting for unequal variances between the groups.

## 3. Results

### 3.1. Descriptive Statistics and Correlational Analysis

Table 2 presents descriptive statistics for the children’s temperamental characteristics and EF measures as an entire group. The overall distributions of the children’s temperament dimensions (rated using a seven-point scale) ranged from 2.0 to 6.92, with the means close to four, except in the case of effortful control. The fact that the mean of effortful control was close to six (M = 5.54) signifies that the participating children were relatively well regulated in their attention and behavioral inhibition. Descriptive statistics of the main variables, based on experimental conditions at the pre- and post-measurement points, are presented in Table 3.

The children’s temperamental surgency and negative emotionality were negatively correlated with each other to a moderate degree (*r* = −0.31, *p* < 0.001); none of the three temperamental sub-dimensions were correlated with the children’s pre- and post-EF scores. The children’s pre- and post-test scores showed relatively strong to strong correlations within the respective tasks: digit span (*r* = 0.53, *p* < 0.001), Corsi block (*r* = 0.49, *p* < 0.001), and DCCS (*r* = 0.43, *p* < 0.001) (Table 1). The pre- and post-cortisol levels were also strongly correlated (*r* = 0.76, *p* < 0.001), meaning that those with higher cortisol levels before VR exposure showed relatively higher levels after VR exposure, as generally observed in non-clinical populations [55,57]. As there were only weak and sporadic correlations among the EF scores across the tasks, the respective tasks were analyzed separately in the main analyses. Only the Corsi block task had a few significant correlations with the digit span and DCCS—and only to a weak degree. There were no significant correlations among the EFs, cortisol, or HRV indices (SDNN, NN50, pNN50, and RMSSD). Next, the family income and the parents’ educational attainment were not significantly correlated with the children’s EF scores, except between the pre-digit span score and family income (*r* = 0.17, *p* < 0.05); they were similarly not correlated with the cortisol levels or HRV indices.

### 3.2. RQ 1.1: Classroom Size (Large vs. Small) and Children’s Performance on EF Tasks

**Figure 3:**
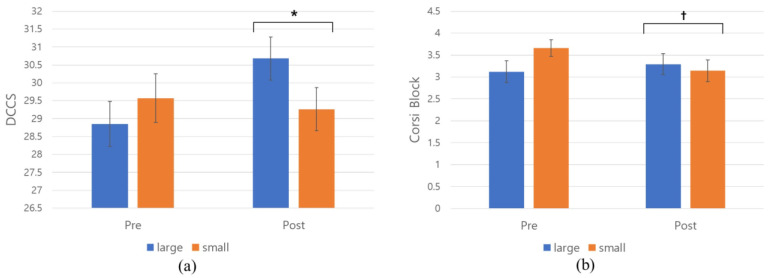
ANCOVA outcomes of the DCCS (**a**) and the Corsi block (**b**) in the classroom size conditions; * *p ≤* 0.05; † *p* ≤ 0.10.

An ANCOVA was run on a sample of 69 participants in the classroom size conditions (n = 34 for the large classroom and n = 35 for the small classroom) to examine the effects of the VR conditions (large vs. small) and pre-EF scores on the post-EF scores (Table 4). Notably, a significant difference emerged in the post-DCCS scores (F(1, 66) = 4.40, *p* = 0.039, ηp^2^ = 0.061), with pre-DCCS scores also demonstrating significance (*p* = 0.001). The results showed that children in the large classroom exhibited significant increase in the DCCS scores than those in the small classroom condition (Figure 3a). Approximately 6% of the variance in the post-DCCS scores could be attributed to the classroom size, indicating a medium effect size [58].

However, when considering other EF measures, no significant effects of classroom size conditions were observed (*p* = 0.68 for the digit span task and *p* = 0.10 for the Corsi block task), with the pre-EF scores being significant. However, it is noteworthy that a marginal difference emerged in the Corsi block task through the ANCOVA (F(1, 66) = 2.71, *p* = 0.10, ηp^2^ = 0.039) (Table 4, Figure 3b). This suggests that approximately 4% of the variance in the post-Corsi block scores could be attributed to the classroom size, representing a small-to-medium effect [58]. Interestingly, this marginal disparity seems linked to *a* decline in test scores among children in the small room condition after VR exposure, as indicated in Table 3 (t = 2.65, *p* < 0.001), whereas those in the large room condition did not exhibit significant pre-to-post score changes.

To summarize, children exposed to the large classroom condition demonstrated notable enhancements in their DCCS task performance, whereas children in the small room condition exhibited a marginally significant reduction in their Corsi block scores. These findings imply a positive influence of larger classroom size on attention and cognitive flexibility, and a potential negative impact of smaller classroom size on working memory.

### 3.3. RQ 1.2: Classroom Size (Large vs. Small) and Children’s Cortisol and HRV

The cortisol levels exhibited a decline in both the large and small classroom conditions, aligning with the anticipated diurnal cortisol rhythm (Table 3). Unexpectedly, the ANCOVA indicated that the classroom size condition did not yield significant effects on the post-cortisol levels, as evidenced by F(1, 66) = 0.01, *p* = 0.929, while the pre-cortisol levels’ significant effects were found (*p* = 0.000) (Table 4).

In relation to the HRV, independent *t*-tests, adjusting unequal variances, unveiled no statistically significant differences in any of the indices between the large and small classroom conditions: SDNN (*t* = −1.32), NN50 (*t* = −1.58), pNN50 (*t* = −1.25), and RMSSD (*t* = −1.25). Nevertheless, it is noteworthy that children in the large classroom condition tended to exhibit higher levels across all indices (Table 3), with two indices displaying marginally significant between-group differences: SDNN (*p* = 0.096) and NN50 (*p* = 0.059). Due to the potential for increased error rates stemming from multiple comparisons, this observation should be approached with caution. That said, these trends could suggest that children in the large classroom condition might have experienced a greater sense of relaxation and reduced stress compared to their peers in the small classroom condition.

### 3.4. RQ 2.1: Window View (Nature vs. Built) and Children’s Performance on EF Tasks

An ANCOVA was conducted on a sample of 72 participants to explore the influence of the window view conditions (nature view (n = 34) vs. built view (n = 38)) and pre-experimental EF scores on the post-experimental scores (Table 5). Contrary to expectations, the ANCOVA indicated that the effects of the window view were not significant on any of the post-EF scores: digit span (F(1, 69) = 0.46, *p* = 0.501), Corsi block (F(1, 69) = 0.52, *p* = 0.474), and DCCS (F(1, 69) = 0.21, *p* = 0.645). Overall, the influence of a window view on the children’s executive functions (EF) was not supported in either of the window view conditions. Contrary to the outcomes reported in prior studies, children in the nature view condition did not display significant improvements in any of the tasks assessing attention and memory.

### 3.5. RQ 2.2: Window View (Nature vs. Built) and Children’s Cortisol and HRV

Against the backdrop of the natural daily cortisol rhythm, a significant reduction in cortisol levels was evident only in the nature view condition between the pre- and post-measurement points. In contrast, there was no significant change in the built view condition (within-group difference) despite a 30 to 40 min passage of time between the two measurement points (Table 3, nature view: *t* = 2.30, *p* = 0.014; built view: *t* = 0.85; *p* = 0.201). Children in the built view condition exhibited relatively lower levels of cortisol at the pre-measurement point than those in the nature view condition, although this between-group difference was not statistically significant. A subsequent ANCOVA revealed that the window view condition had no significant impact on the post-cortisol levels (F(1, 69) = 0.31, *p* = 0.576), while effects were pronounced on the pre-cortisol levels (*p* = 0.000) (Table 5).

Next, in relation to the HRV, independent *t*-tests with adjusted unequal variances unveiled no statistically significant differences in any of the indices between the natural and built view conditions: SDNN (*t* = −0.05), NN50 (*t* = 0.79), pNN50 (*t* = 0.51), and RMSSD (*t* = 0.13). Unlike the classroom size conditions, no consistent tendencies or significant differences were observed in any of the HRV indices between these two groups.

## 4. Discussion

This study aimed to investigate the impact of classroom size (large vs. small) and window view (nature vs. built) on five-and six-year-old children’s EF performance using VR technology and all significant changes in cortisol and HRV as physiological stress indices. Contrary to the initial hypotheses regarding RQ2, the findings of this study do not strongly support the notion that the window view of an early childhood education and care (ECEC) classroom significantly influences young children’s executive functions and stress responses (cortisol and HRV). However, notable results emerged in the classroom size conditions (RQ 1.1). Consistent with the initial hypothesis, children assigned to the large classroom condition exhibited significant improvements in their post-DCCS task performance, while controlling for their initial DCCS performance. In contrast, those in the small classroom condition did not display the same level of enhancement. Conversely, children exposed to the small classroom environment exhibited marginally significant decrease in the post-experimental Corsi block task, while their counterparts in the large classroom did not demonstrate any significant changes in their scores. As for stress response indicators, the study found no significant impact from either the classroom size or the window view conditions (RQ 1.2). However, it was worth noting that the children in the large classroom condition showed marginally significant higher values in certain HRV indices compared to those in the small classroom condition.

### 4.1. The Impact of Classroom Size and Window View on Young Children’s EFs

The study findings regarding the classroom size conditions are in line with a previous study, which found that spatial density in the classroom had a negative effect on students’ academic achievement [31]. Moreover, the observed pattern where children in the large classroom group consistently exhibited higher HRV indices compared to their counterparts in the small classroom group indicates a potential increase in relaxation among children in the larger classroom condition, aligning with the initial hypothesis. However, it is important to note that the magnitude of these effects did not attain traditional levels of statistical significance.

Then, why was attentional flexibility positively impacted by a large classroom while not being adversely affected by a small classroom? Possibly, attentional control could be more readily influenced by physiological relaxation, rather than stressors. In fact, a few studies have supported this possibility. Even though not many studies have focused on comparing the impact of positive and negative physiological or emotional states on cognitive flexibility, there have been a few [59,60,61]. For instance, positive emotions (i.e., amusement and contentment) had advantageous effects on college students’ attention and cognitive flexibility by broadening attentional focus and facilitating the integration of diverse information, while negative emotions (i.e., anger and anxiety) had only a very weak impact (null finding in the first experiment and a marginally significant finding in the second experiment). These study findings make sense in terms of the evolutionary benefits that positive emotions can bring to survival and prosperity by boosting cognitive resources for continuous well-being and flourishing, while unfavorable impacts of negative emotions should be kept minimal to prevent and cope with danger [59,62]. However, it should be noted that evidence suggests that attentional flexibility can be influenced by stressful situations [63], necessitating further investigation.

Similarly, regarding the significant decline in visuo-spatial working memory within the small classroom condition, there has been no research into whether visuo-spatial working memory is more vulnerable than attentional control to stressful circumstances. Currently, evidence indicates that both visuo-spatial working memory and attentional flexibility can be affected by acute stressors [63,64]. Thus, there is a gap in knowledge when it comes to comparing their individual susceptibilities. Delving into the intricacies of these relationships is crucial for a deeper comprehension of how diverse cognitive functions are differentially impacted by stress and external factors.

Next, in relation to the observation that the adverse effect of a small classroom was evident solely in visuo-spatial working memory, not in verbal working memory, a possible explanation may be tied to the distinct neural pathways associated with the operation of these two working memory types—referred to as the visuo-spatial sketchpad for the former and the phonological loop for the latter [65]. Several neuroimaging studies have suggested that the visuo-spatial sketchpad is associated with a broader network of brain structures compared to the phonological loop [66,67]. This difference is likely due to the fact that visuo-spatial tasks entail not only memory storage but also the manipulation of visual and spatial information. Given that the manipulating visual–spatial information involves the engagement of a greater number of brain regions, the functioning of the visuo-spatial sketchpad is more likely to be susceptible to the effects of stress. Therefore, in the context of the present study, it is plausible that the children’s performance decline on the task assessing visuo-spatial working memory occurred, as opposed to verbal working memory. Indeed, a few prior studies have documented that visuo-spatial working memory exhibits greater susceptibility to stressful situations (such as academic stress and anxiety) compared to verbal working memory [68,69].

Shifting attention to the natural and built view conditions, no significant impact of either condition was observed across all post-EF tasks and physiological indices. These findings stand in contrast to the attention restoration theory [17] and the stress reduction theory [19], as well as relevant prior research that has highlighted the beneficial influence of natural environments on cognitive and emotional functioning [8,9,21,23]. These unexpected outcomes may have arisen due to the following reasons. First, children in the window view condition might not have allocated sufficient attention to the various window views during the virtual reality (VR) experience. Unlike classroom size, which can evoke automatic responses without conscious effort, the impact of the window view requires participants’ visual attention to the treatment variable in order to yield either positive or negative effects on their cognition and emotions. However, in the experimental procedures employed in this study, the children were not specifically instructed to focus on the windows. This decision was intended to simulate a scenario closer to their typical daily experiences. Considering that the realistic VR environment was highly captivating and intriguing for young children (as evident from their verbal responses during VR exposure), it is plausible that they may not have dedicated sufficient attention to the window views. If this indeed influenced the results, future studies should consider modifying the procedures to include explicit instructions for participants to actively attend to the window views for a meaningful duration.

Expanding on the aforementioned point, another plausible explanation for the absence of significant findings in the window view condition could be attributed to the relatively short ten-minute duration of VR exposure. While the choice of exposure time was informed by previous studies primarily involving adult participants, it is plausible that a longer exposure period might have been necessary to evoke stress reduction responses among young children in the VR environment. Furthermore, it is plausible that preschool-aged children, who are yet to begin formal education, typically experience lower baseline levels of stress and have less cognitively demanding tasks related to attention and memory. In contrast to older students, whose learning environments are often academically rigorous and can induce stress, preschool curricula are crafted to balance play, learning, and rest, prioritizing sensory exploration and social interactions. Consequently, for preschoolers, the classroom may not be associated with the same level of stress and demand for sustained attention and self-control as it is for secondary or upper-level students. This distinction in perception and experience could potentially diminish the apparent benefits of a natural view in preschool settings. A study involving fourth graders [55], slightly older than the participants in the current study, revealed that nature views did not exert any influence on attention or concentration scores, contrary to findings among older high-school or college students [8,9,21]. Therefore, further investigation is warranted to uncover which factors (such as gaze duration toward the window view, participant age, exposure duration, etc.) might elucidate these discrepant outcomes.

### 4.2. Physiological Responses in Relation to VR Conditions and Children’s EFs

No significant impact of the classroom size and window view were found on the physiological signals of stress reduction and resilience in either VR condition. The results regarding the window view condition is against the stress reduction theory (SRT), which argues that exposure to nature can promote relaxation and restoration, leading to decreased levels of physiological arousal and stress-related hormones [19]. In relation to the large vs. small classroom conditions, however, the between-group differences in SDNN and NN50 were marginally significant, with the large classroom group showing higher values. Also, all other relevant indices tended to be higher (although non-significant) among children in the large classroom condition. These results suggest that the large-classroom children may have become slightly more relaxed and less physically stressed than the small-classroom children through virtual exposure to a spacious classroom, even though no degree of significance was detected. Possibly, a larger sample size of children, in conjunction with the factors mentioned earlier (such as an extended exposure duration, increased attention to the treatment variable, etc.), could be necessary to detect clearer and more robust significant effects.

Unlike previous studies, which have found significant associations between acute stress and changes in cortisol and EFs (i.e., working memory measured using Corsi block and digit span and cognitive flexibility measured using DCCS) [70] and between HRV and EFs [45,46], there were no significant correlations between cortisol, HRV, and EFs in the current study. Generally, cortisol responses peak around 20 to 30 min after the onset of a stressor due to the activation of the hypothalamic–pituitary–adrenal (HPA) axis, which releases cortisol as part of the body’s stress response [71]. Given that the post-experimental cortisol was measured after the completion of the EF tasks, which took place approximately 15 min after VR exposure, this might have been a possible cause of no correlation between cortisol and EFs in the present study.

Generally, executive functions (EFs) and heart rate variability (HRV) are closely interconnected [45,46]. This connection is evident in the brain regions implicated in both executive functioning and the modulation of the autonomic nervous system (ANS), such as the prefrontal cortex [72]. Despite the established connections, the absence of significant associations between HRV and EFs might be attributable to inadequate fluctuations in stress levels induced by the VR conditions. Acute stress prompts the activation of the sympathetic nervous system (SNS) within the ANS, resulting in cortisol secretion [73]. Elevated cortisol levels during stress can have adverse impacts on the prefrontal cortex—a pivotal brain region for executive functions [74]. In this context, if the VR experiences in the current study did not evoke substantial autonomic responses—possibly due to the brief exposure duration or limited attention to the treatment variable—they might not have induced significant changes in both HRV and EFs, leading to the observed lack of associations between them. Moreover, it is also conceivable that the five-minute duration might not have been adequate to capture HRV changes in the young children, contrary to the findings from adult participants. To address this possibility, a potential solution could be extending the HRV data collection time by modifying the experimental procedures—for instance, allowing the children to remain seated for a longer period before enabling movement. Such modifications could align the future study’s outcomes more closely with prior research findings.

### 4.3. Limitations and Future Studies

This study possesses unique strengths, as it pioneers the exploration of spatial elements’ impacts on young children’s cognitive and physiological functioning in ECEC settings. Utilizing VR technology and grounded in a robust randomized experimental study design, it stands as the inaugural research in this domain. Nevertheless, there remains scope for further refinement in its experimental design and measurement techniques. Firstly, it could be beneficial to incorporate EF tests within virtual contexts, akin to the approach undertaken by Cruz-Garza et al. (2022) [28], thus enabling a more comprehensive exploration of valid effects. In addition, it is important to acknowledge that the EF tasks were administered in the same order to all participants and thus might have introduced order effects to the outcomes. Thus, subsequent research endeavors should consider these methodological adaptations.

Furthermore, a notable aspect lacking in the current study is the control for participants’ exposure to nature within their daily lives, the size of their home environments, and the characteristics of their ECEC classrooms in statistical analyses. Given the established associations between connections to nature, home crowding, and students’ academic achievements [31,75,76], future investigations would gain by factoring in these variables. Addressing the same thread, variations between the experimental and comparison groups in the VR classroom environments beyond the designated treatment variable should be addressed in future studies. Particularly, factors like window sizes and proportions to the wall were dissimilar between the large and small classrooms, which might have potentially impacted the study results.

Subsequently, this study aimed to assess the individual impacts of class size and window view. In future research, investigating the combined effects of both variables on children’s developmental outcomes would yield valuable insights. Additionally, the virtual classroom in this study featured a light blue wall color. Given the documented influence of colors on cognitive functioning [6,77], it becomes essential to explore how varying wall colors in virtual environments could potentially affect outcomes.

It is worth noting that the experiment took place amid the COVID-19 pandemic, posing considerable challenges in participant recruitment. Despite these challenges, the sample size for each experiment and comparison group in this study exceeded those in related experimental studies, such as Li and Sullivan (2016) [9] and Palanica et al. (2019) [10]. However, our focus on young children suggests that an even larger sample might have been advantageous, allowing for a more detailed exploration of the nuanced impact of spatial factors on this age group.

## 5. Conclusions

The design of educational environments holds the power to significantly influence students’ cognitive and socioemotional development. Concepts such as ensuring adequate personal space per student and providing a window view of nature have long been associated with enhancing cognitive and emotional functioning by reducing stress and facilitating rapid recovery. In partial alignment with prior research, the findings of the current study suggest a potential positive impact of ample classroom space on the attentional flexibility and working memory of pre-primary children. Also, no prior studies to date have effectively disentangled the influence of classroom size from the confounding effects of crowding. In this context, the findings of the present study beckon further inquiry into the intricate relationships between classroom size and children’s developmental outcomes. Recognizing the acknowledged detrimental impact of social density on students’ cognition and behavioral adaptation, along with the undeniable significance of available space per child in determining classroom size, it becomes evident that the pursuit of an optimal synergy between appropriate social density and spatial density warrants deeper exploration. The study outcomes, coupled with the scarcity of pertinent investigations focusing on young children, underscore the urgent need for more research and heightened emphasis on understanding the influence of spatial factors on young learners in early childhood education and care (ECEC) settings. As an initial stride in this direction, the present study emphasizes the significance of offering sufficient classroom space to young children within ECEC settings. Further research in this realm could lead to the formulation of appropriate standards for ECEC classroom dimensions and their legal incorporation.

## Figures and Tables

**Table 1:** Correlations among the main and socio-demographic variables.

	1	2	3	4	5	6	7	8	9	10	11	12	13	14	15	16	17	18
1. Surg	−	−	−	−	−	−	−	−	−	−	−					−	−	−
2. NE	−0.31 ***	−	−	−	−	−	−	−	−	−	−					−	−	−
3. EC	0.04	−0.15	−	−	−	−	−	−	−	−	−					−	−	−
4. Pre-DS	−0.09	−0.03	0.10	−	−	−	−	−	−	−	−					−	−	−
5. Post-DS	−0.11	0.02	−0.02	0.53 ***	−	−	−	−	−	−	−					−	−	−
6. Pre-CB	−0.11	0.06	−0.10	0.08	0.11	−	−	−	−	−	−					−	−	−
7. Post-CB	−0.28	0.05	0.07	0.12	0.18 *	0.49 ***	−	−	−	−	−					−	−	−
8. Pre-DCCS	0.07	−0.08	−0.02	0.09	0.03	0.19 *	0.18 *	−	−	−	−					−	−	−
9. Post-DCCS	0.08	−0.12	0.04	0.06	0.00	0.17 *	0.08	0.43 ***	−	−	−					−	−	−
10. Pre-Corti	0.07	0.13	0.01	0.15	0.11	0.02	0.01	0.11	0.00	−						−	−	−
11. Post-Corti	−0.04	0.05	0.05	0.14	0.07	−0.01	−0.01	0.10	0.02	0.76 ***	−					−	−	−
12. SDNN	−0.04	−0.07	0.05	0.07	0.11	−0.05	0.03	0.00	−0.14	0.18 *	0.04							
13. NN50	0.03	−0.13	0.07	0.09	0.07	−0.14	−0.08	−0.13	−0.09	0.06	−0.00	0.70 ***						
14. pNN50	0.04	−0.01	0.11	0.06	0.03	−0.08	−0.03	−0.13	−0.05	0.13	0.04	0.73 ***	0.82 ***					
15. RMSSD	−0.03	−0.04	0.05	0.05	0.09	−0.04	0.04	0.01	−0.13	0.19	0.04	0.99 ***	0.65 ***	0.73 ***				
16. FE	0.03	−0.08	−0.04	0.09	−0.03	0.04	0.01	0.04	0.05	0.07	0.10	0.14	0.09	0.06	0.12	−	−	−
17. ME	0.06	0.04	−0.08	0.16	0.13	−0.01	0.02	−0.02	0.01	0.11	0.08	0.00	−0.04	−0.02	0.00	0.42 ***	−	−
18. Income	−0.05	−0.11	0.01	0.17 *	0.16+	−0.01	0.11	−0.01	−0.00	0.25 **	0.14	0.05	0.01	0.02	0.04	0.26 **	0.29 ***	−
19. Child sex	−0.00	0.00	0.03 ***	0.00	0.01	−0.02	0.12	−0.06	0.11	0.11	0.03	0.12	0.08	0.21 *	0.13	0.04	0.09	0.20 *

Note.: pre- = pre-VR exposure; post- = post-VR exposure; Surg = surgency (factor score); NE = negative emotionality; EC = effortful control; DS = digit span; CB = Corsi block; DCCS = dimension change card sorting; Corti = cortisol; FE = father’s education; ME = mother’s education; Income = family income; Child sex: 1 = boys, 2 = girls. * *p ≤* 0.05, ** *p ≤* 0.01, *** *p ≤* 0.001.

**Table 2:** Descriptive statistics for children’s temperament and executive functions as an entire group.

	N	M	SD	Min	Max
Temperament					
Surgency	141	4.15	0.75	2.33	6.42
Negative emotionality	141	3.86	0.67	2.00	5.67
Effortful control	141	5.54	0.66	3.25	6.92
Executive Functions					
Digit span (pre)	141	3.50	0.95	0	6
Digit span (post)	141	3.67	0.97	2	7
Corsi block (pre)	141	3.18	1.32	0	6
Corsi block (post)	141	3.06	1.43	0	7
DCCS (pre)	141	28.68	3.92	15.00	36.00
DCCS (post)	141	29.74	3.66	20.00	36.00

Note. pre = pre-VR exposure; post = post-VR exposure.

**Table 3:** Descriptive statistics for children’s executive functions, cortisol levels, and heart rate variability by experimental conditions (n = 141).

		Room Size		Window View	
		Large (n = 34)	Small (n = 35)	Nature (n = 34)	Built (n = 38)
		M (SD)	M (SD)	M (SD)	M (SD)
Digit Span	Pre	3.67 (0.68)	3.57 (0.88)	3.50 (1.05)	3.26 (1.08)
	Post	3.79 (0.97)	3.65 (0.93)	3.76 (1.13)	3.50 (0.86)
Corsi block	Pre	3.12 (1.45)	3.66 (1.13)	3.18 (1.35)	2.79 (1.21)
	Post	3.29 (1.40)	3.14 (1.42)	3.14 (1.37)	2.71 (1.50)
DCCS	Pre	28.85 (3.70)	29.57 (4.05)	28.26 (4.26)	28.08 (3.67)
	Post	30.68 (3.51)	29.26 (3.57)	29.76 (3.54)	29.32 (3.97)
Cortisol	Pre	7.19 (3.55)	6.47 (2.77)	6.49 (2.83)	5.83 (2.48)
	Post	6.21 (2.45)	5.77 (2.01)	5.81 (2.32)	5.55 (3.01)
HRV	SDNN (ms)	127.78	95.21	98.63	99.75
	NN50 (ms)	39.88	31.55	34.17	30.62
	pNN50 (%)	0.19	0.16	0.17	0.16
	RMSSD (ms)	164.62	121.09	124.40	120.73

Note. M = mean; SD = standard deviation; pre = pre-VR exposure; post = post-VR exposure.

**Table 4:** Results of one-way ANCOVA for the classroom size condition.

Source	Sum of Squares	df	Mean Square	*F*-Value	*p*-Value	ηp^2^
Dependent variable: Post-digit span						
Model	9.46	2	4.73	5.97	0.00 **	0.15
Classroom size	0.13	1	0.13	0.17	0.68	0.00
Pre-digit span	9.13	1	9.14	11.53	0.00 **	0.15
Residual	52.31	66	0.79			
Total	61.77	68	0.91			
Dependent variable: Post-Corsi block						
Model	33.25	2	16.63	9.92	0.00 ***	0.23
Classroom size	4.55	1	4.55	2.71	0.10 ^†^	0.04
Pre-Corsi block	32.28	1	32.28	19.25	0.00 ***	0.23
Residual	110.66	66	1.68			
Total	143.91	68	2.12			
Dependent variable: Post-DCCS						
Model	157.49	2	78.74	7.26	0.00 **	0.18
Classroom size	47.66	1	47.66	4.40	0.04 *	0.06
Pre-DCCS	122.74	1	122.74	11.32	0.00 **	0.15
Residual	715.38	66	10.84			
Total	872.87	68	12.84			
Dependent variable: Post-cortisol						
Model	220.32	2	110.16	61.21	0.00 **	0.65
Classroom size	0.014	1	0.01	0.01	0.93	0.00
Pre-cortisol	217.08	1	217.08	120.62	0.00 ***	0.65
Residual	118.78	66	1.80			
Total	339.10	68	4.99			

Note. * *p ≤* 0.05, ** *p ≤* 0.01, *** *p ≤* 0.001, ^†^ *p* ≤ 0.10.

**Table 5:** Results of one-way ANCOVA for the window view condition.

Source	Sum of Squares	df	Mean Square	*F*-Value	*p*-Value	ηp^2^
Dependent variable: Post-digit span						
Model	28.92	2	14.46	23.77	0.00 ***	0.41
Window view	0.28	1	0.28	0.46	0.50	0.01
Pre-digit span	27.66	1	27.66	45.48	0.00 ***	0.40
Residual	41.96	69	0.61			
Total	70.88	71	1.00			
Dependent variable: Post-Corsi block						
Model	40.97	2	20.49	13.03	0.00 ***	0.27
Window view	0.82	1	0.82	0.52	0.47	0.01
Pre-Corsi block	37.57	1	37.57	23.89	0.00 ***	0.26
Residual	108.51	69	1.57			
Total	149.50	71	2.11			
Dependent variable: Post-DCCS						
Model	245.47	2	122.73	11.22	0.00 ***	0.25
Window view	2.35	1	2.35	0.21	0.64	0.00
Pre-DCCS	241.85	1	241.85	22.12	0.00 ***	0.24
Residual	754.48	69	10.93			
Total	999.94	71	14.08			
Dependent variable: Post-cortisol						
Model	284.73	2	142.36	43.09	0.00 ***	0.66
Window view	1.04	1	1.04	0.32	0.58	0.08
Pre-cortisol	283.52	1	283.52	85.81	0.00 ***	0.66
Residual	227.98	69	3.30			
Total	512.71	71	7.22			

Note. *** *p ≤* 0.001.

## Data Availability

The data that support the findings of this study are available from the author upon reasonable request.

## Supplementary Materials

The following supporting information can be downloaded at https://www.mdpi.com/article/10.3390/bs13110936/s1: Figure S1: Floor plans for VR conditions.
