# Supplementary material for: The Influence of Classroom Size and Window View on Young Children’s Executive Functions and Physiological Responses, Based on VR Technology
Source: Behav Sci (Basel). 2023 Nov 16;13(11):936. doi: 10.3390/bs13110936 (PMC10668947; doi:10.3390/bs13110936)
Supplement: Supplementary file 1 [file behavsci-13-00936-s001.zip › behavsci-2718733-supplementary.pdf]

Supplementary material 1. Floor plans for VR conditions: (a) large classroom (also for nature- and built-view conditions); (b): small classroom

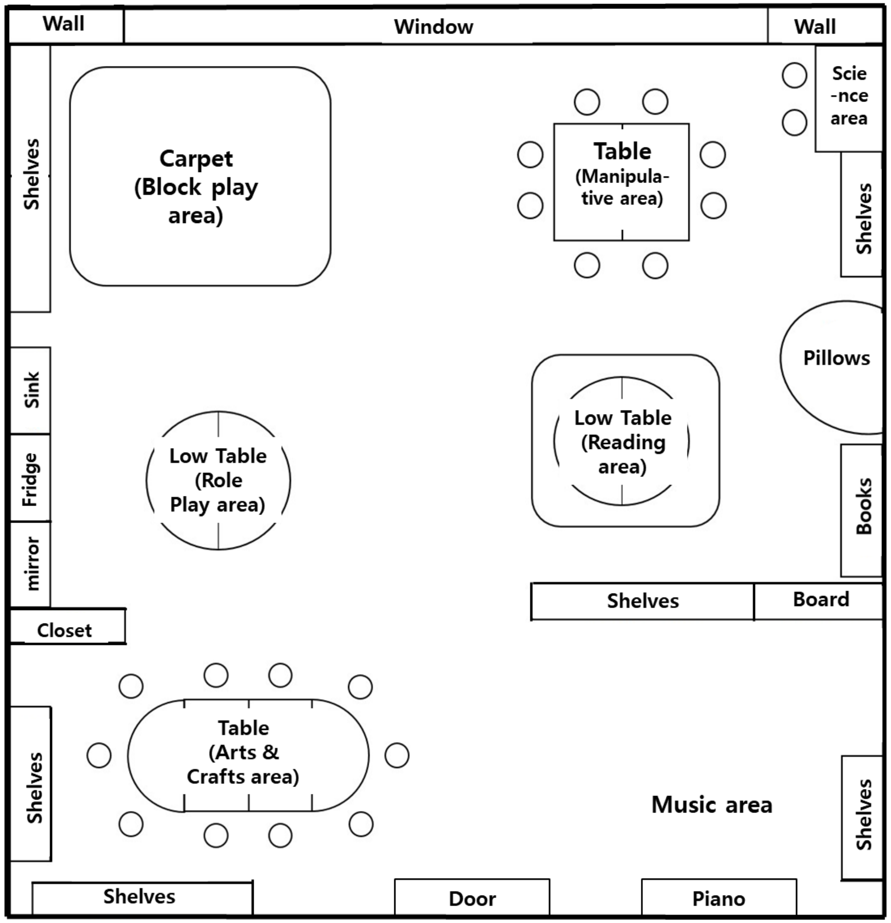

(a)

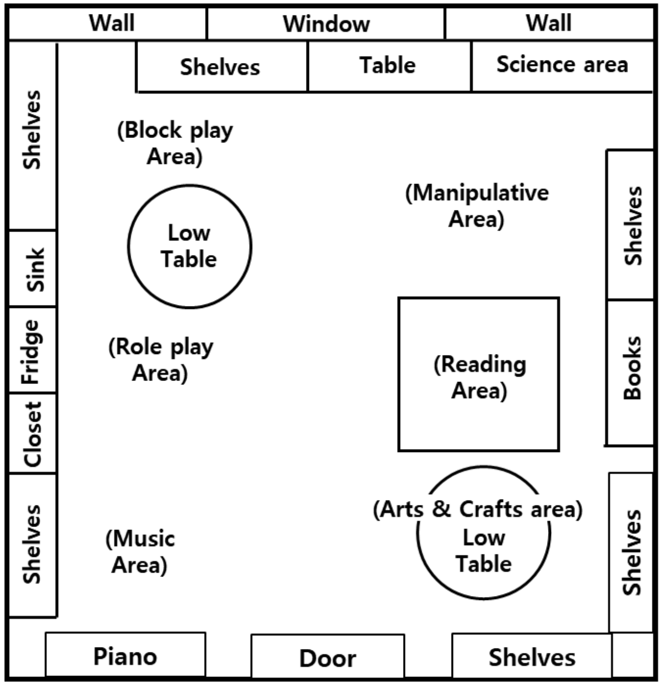

(b)
